# An improved method of constructing degradome library suitable for sequencing using Illumina platform

**DOI:** 10.1186/s13007-019-0524-7

**Published:** 2019-11-18

**Authors:** Yong-Fang Li, Miao Zhao, Menglei Wang, Junqiang Guo, Li Wang, Jie Ji, Zongbo Qiu, Yun Zheng, Ramanjulu Sunkar

**Affiliations:** 1 College of Life Sciences, Henan Normal University, Xinxiang, Henan People’s Republic of China; 2 Faculty of Information Engineering and Automation, Kunming University of Science and Technology, Kunming, 650500 Yunnan China; 3 Yunnan Key Laboratory of Primate Biomedical Research, Institute of Primate Translational Medicine, Kunming University of Science and Technology, Kunming, 650500 China; 4 Department of Biochemistry and Molecular Biology, Oklahoma State University, Stillwater, OK 74078 USA

**Keywords:** Cleavage, Degradome, Illumina sequencing, miRNA, Target gene

## Abstract

**Background:**

Post-transcriptional gene regulation is one of the critical layers of overall gene expression programs and microRNAs (miRNAs) play an indispensable role in this process by guiding cleavage on the messenger RNA targets. The transcriptome-wide cleavages on the target transcripts can be identified by analyzing the degradome or PARE or GMUCT libraries. However, high-throughput sequencing of PARE or degradome libraries using Illumina platform, a widely used platform, is not so straightforward. Moreover, the currently used degradome or PARE methods utilize MmeI restriction site in the 5′ RNA adapter and the resulting fragments are only 20-nt long, which often poses difficulty in distinguishing between the members of the same target gene family or distinguishing miRNA biogenesis intermediates from the primary miRNA transcripts belonging to the same miRNA family. Consequently, developing a method which can generate longer fragments from the PARE or degradome libraries which can also be sequenced easily using Illumina platform is ideal.

**Results:**

In this protocol, 3′ end of the 5′RNA adaptor of TruSeq small RNA library is modified by introducing EcoP15I recognition site. Correspondingly, the double-strand DNA (dsDNA) adaptor sequence is also modified to suit with the ends generated by the restriction enzyme EcoP15I. These modifications allow amplification of the degradome library by primer pairs used for small RNA library preparation, thus amenable for sequencing using Illumina platform, like small RNA library.

**Conclusions:**

Degradome library generated using this improved protocol can be sequenced easily using Illumina platform, and the resulting tag length is ~ 27-nt, which is longer than the MmeI generated fragment (20-nt) that can facilitate better accuracy in validating target transcripts belonging to the same gene family or distinguishing miRNA biogenesis intermediates of the same miRNA family. Furthermore, this improved method allows pooling and sequencing degradome libraries and small RNA libraries simultaneously using Illumina platform.

## Background

The regulation of gene expression is controlled at multiple levels and the mRNA degradation/decay is one of the important determinants in this process. The mRNA degradation pathway is highly conserved in eukaryotes, and is controlled by exonucleases that can cause either 5′ to 3′ or 3′ to 5′ decay [1–4]. In addition, endonuclease-dependent mRNA degradation which is guided by the small RNAs (miRNAs or siRNAs) emerged as yet another important conserved mRNA degradation pathways in higher eukaryotes [5, 6]. Plant miRNAs can cause degradation of the target mRNAs primarily by Argonaute (endonuclease)-mediated cleavage within the target site leaving a monophosphate at the 5′end of the 3′cleaved mRNA fragment [7, 8]. Because plant miRNAs can target mRNAs that possesses perfect or near-perfect complementarity, their targets can be largely predicted using computational approaches [9, 10]. However, false positive rate in such target predictions is high, therefore experimental validation is necessary. Modified 5′ RACE (Rapid Amplification of cDNA Ends) is widely used technique to map in vivo cleavage sites induced by miRNA [11]. However, this approach is time consuming, labor-intensive and costly. To overcome these limitations, methods such as the PARE (parallel analysis of RNA ends) [12, 13], degradome [14] and GMUCT (genome-wide mapping of uncapped and cleaved transcripts) [15] that combine the 5′RACE and high throughput sequencing of short reads have been developed. GMUCT technique generates variable length fragments for sequencing [15, 16], while, both PARE and degradome take advantage of MmeI digestion to generate consistent sized fragment (20-nt) (named as “tag” or “signature”) derived from the 5′end of 3′cleaved product [8, 13, 14]. Detailed methodology of generating PARE or degradome libraries have been reported previously [12, 17]. Moreover, by incorporating index into the library construction that allows multiplexing of degradome libraries for Illumina HiSeq sequencing, the PARE or degradome library construction has been further improved [18]. However, sequencing of the degradome or PARE library in an Illumina sequencer is complicated to some degree and not as straightforward as sequencing of other TruSeq libraries such as the small RNA library. This is due to the fact that 5′RNA adaptor length is varied between these two different libraries, i.e., the 5′RNA adaptor (RA5) of small RNA library is slightly longer than that of degradome or PARE library. Therefore, a specific PARE sequencing primer has to be used for sequencing. Regrettably, this sequencing primer is not compatible with the standard Illumina TruSeq sequencing primer, thus ‘‘SR_TubeStripHyb’’ manual must be used during cluster generation [18]. Another notable drawback with the currently-used degradome or PARE protocols is that these libraries yield reads or tags that are only 20-nt long, which poses difficulty in distinguishing between the members of the same target gene family.

**Fig. 1:**
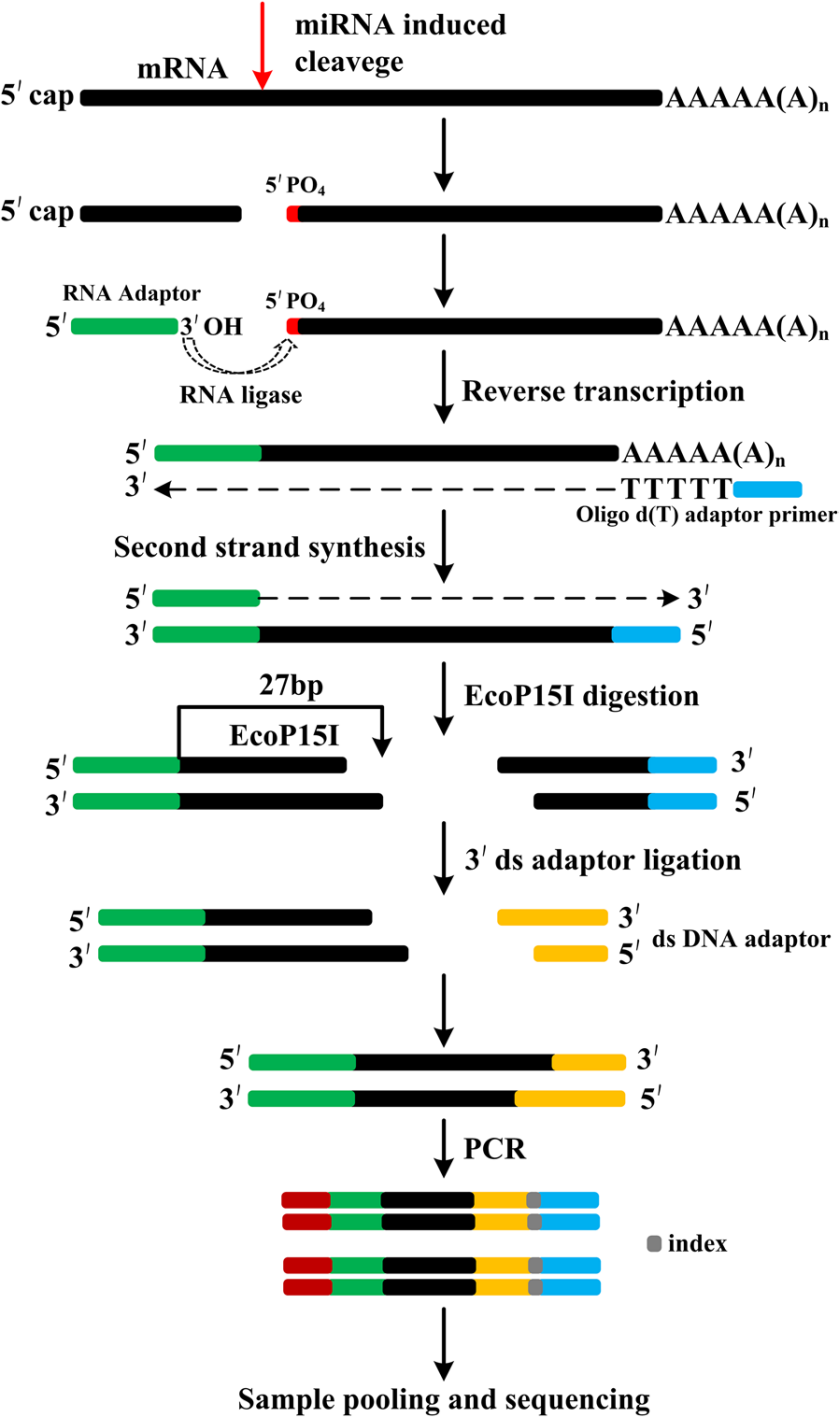
The scheme for constructing improved degradome library. For sequencing purposes, the degradome library generated by this method can be treated as small RNA library and the resulting reads are ~ 27 nt long. The procedure includes: (1) poly(A) RNA isolation; (2) 5′RNA adapter ligation to uncapped poly(A) RNA with 5′ monophosphate; (3) reverse transcription to generate 1st strand cDNA using an oligo(dT)-tailed adapter (RT-primer); (4) second strand synthesis (1st PCR amplification); (5) EcoP15I digestion to generate ~ 27 nt long reads; (6) ligation of EcoP15I digestion products with a 3′ds-DNA adapter; (7) purification of ligation products on a PAGE gel; (8) degradome library enrichment (2nd PCR amplification); (9) purification of the final product on a PAGE gel; (10) library pooling and sequencing using Illumina HiSeq platform

Besides identifying miRNA targets, degradome or PARE libraries have the potential to reveal miRNA biogenesis [8, 13, 19]. The degradome tag analysis was instrumental in revealing the loop-first processing of MIR319 hairpins in plants [19]. However, surprisingly a significant number of degradome reads obtained from Arabidopsis [13], rice [8], *Physcomitrella patens* [19] and mouse [20] correspond to mature miRNAs suggesting that some of the miRNAs have been captured in degradome libraries. This could be due to adenylation of the mature miRNAs [21], or incomplete DCL1 cleavage (cleavage only at one arm of the hairpin of pri-miRNA), or loop-first cleavage during miRNA processing. This perplexity is largely due to similar size between mature miRNA reads and degradome reads. Therefore, generation of PARE or degradome tags longer than the length of canonical miRNA/miRNA* will not only improve accuracy in identifying miRNA targets but also in distinguishing between mature miRNA reads versus degradome reads. Additionally, the longer degradome read length can help in understanding the process of miRNA biogenesis. Although a restriction enzyme (EcoP15I) that can generate ~ 27-nt long reads was previously used in degradome libraries, the developed method was suitable for sequencing using Applied Biosystems SOLiD sequencing platform [19]. Given the advantages of Illumina sequencing, a detailed methodology that combines the use of EcoP15I and Illumina HiSeq sequencing platform is ideal. Indeed Zhai et al. [18] has modified the degradome protocol to suit to Illumina HiSeq platform but again MmeI restriction site was used in the RNA adapter. In this improved degradome or PARE protocol, longer read lengths are generated by using EcoP15I and the resulting libraries can be sequenced easily using Illumina sequencer (Fig. 1). Using this improved method, we have successfully constructed and sequenced degradome libraries from rice samples.

## Materials

### Reagents

- TRIzol^®^ reagent (ThermoFisher, Cat. No. 15596-026)
- Dynabeads^®^ mRNA purification kit (ThermoFisher, Cat. No. 610-06)
- 3 M sodium acetate (NaOAc), pH = 5.5, RNase-free (ThermoFisher, Cat. No. AM9740)
- Primers from TruSeq^®^ small RNA sample prep kit-Set A (Illumina, Cat. No. RS-200-0012)
- T4 DNA ligase (ThermoFisher, Cat. No. 15224-017)
- T4 RNA ligase (NEB, Cat. No. M0204S)
- EcoP15I (NEB, Cat. No. R0646S)
- Platinum^®^ Taq DNA polymerase high fidelity (ThermoFisher, Cat. No. 11304011)
- 20 bp DNA ladder (Takara, Cat. No. 3420A)
- 50 bp DNA ladder (Takara, Cat. No. 3421A)
- DL 1000 DNA ladder (Takara, Cat. No. 3591A)
- SuperScript™ II reverse transcriptase (ThermoFisher, Cat. No. 18064)
- RNaseOUT™ recombinant ribonuclease inhibitor (ThermoFisher, Cat. No. 10777-019)
- 40% Acrylamide/Bis19:1 40% (w/v) solution (ThermoFisher, Cat. No. 9022)
- SYBR™ gold nucleic acid gel stain (ThermoFisher, Cat. No. S11494)
- Ethidium bromide solution (Promega, Cat. No. H5041)
- DEPC-treated water (ThermoFisher, Cat. No. AM9906)
- Glycogen (ThermoFisher, Cat. No. 10814-010)
- MinElute^®^ PCR purification kit (QIAGEN, Cat. No. 28004)
- Corning_Costar_Spin-X_centrifuge tube filters (Sigma, Cat. No. CLS8162-24EA)

### Equipments

- Mini-protean tetra cell 4-gel vertical electrophoresis system (Biorad, Cat. No. 165-8001)
- DynaMag™-2 magnet (Thermo Fisher Scientific, Cat. No. 12321D)
- NanoDrop One microvolume UV–vis spectrophotometer (Thermo Fisher Scientific, Cat. No. ND-ONE-W)

### Adapter and primer sequence

- 5′ RNA adapter: 5′-GUUCAGAGUUCUACAGUCCGACGAUC***AGCAG***-3′ [this is the sequence of the 5′RNA adaptor (RA5) of small RNA library with addition of AGCAG at 3′end (bold and italic), which generate the recognition site of EcoP15I (underlined)].
- RT-primer: 5′-CGAGCACAGAATTAATACGACTTTTTTTTTTTTTTTTTTV-3′
- 5′ adaptor primer: 5′-GTTCAGAGTTCTACAGTCCGAC-3′
- 3′ adaptor primer: 5′-CGAGCACAGAATTAATACGACT-3′
- dsDNA_top: 5′-NNTGGAATTCTCGGGTGCCAAGG-3′ (PAGE purified)
- dsDNA_bottom: 5′-CCTTGGCACCCGAGAATTCCA-3′ (PAGE purified)
- Final 5′PCR primer: RP1 from TruSeq^®^ Small RNA Sample Prep Kit
- Final 3′PCR primer: indexed TruSeq 3′ PCR primers, RPI1-12

## Protocol

### Total RNA sample preparation

The total RNA from plant tissues can be isolated using standard RNA isolation kits. We used TRIzol^®^ Reagent for isolating total RNA from rice seedling [17]. Briefly, 0.2 mg tissue was ground to fine powder and homogenized with 4 ml of TRIzol^®^ Reagent; after 5 min incubation at room temperature, 0.8 ml chloroform was added and mixed well; following centrifugation, the upper aqueous phase was transferred to a new tube, and 2 ml isopropanol was added to precipitate RNA; following centrifugation and 75% ethanol washing, RNA pellet was dissolved in DEPC H_2_O. RNA quality and integrity are critical to the success of degradome libraries construction, which can be assessed by running on an agarose gel, using a Nanodrop spectrophotometer or Agilent’s Bioanalyzer. RNA integrity can be checked by electrophoresis on a 1% agarose gel. Using Nanodrop, RNA concentration can be checked, and contaminations in RNA samples can be indicated by A260/280 and A260/230 ratios, which should be close to 1.8 and 2.0, respectively. If using a Bioanalyzer, RNA with high Integrity Number (RIN > 8.0) score is preferred (RIN score ranges from 1 to 10 and RIN 10 indicates highly intact RNA).

### Day 1

#### Poly(A) RNA purification

We use the ThermoFisher Dynabeads mRNA Purification Kit to purify poly(A) RNA, but other mRNA purification kits should work as well. The initial amount of total RNA can be varied from 30 to 200 μg, and usage of higher quantities of initial total RNA will reduce the number of PCR cycles during enrichment of the final degradome library. We used 100 μg, and the volume of reagents and Dynabeads™ magnetic beads for poly(A) RNA purification were scaled based on the instructions (Thermofisher).

1. Initial RNA preparation: adjust the RNA volume to 135 μl with DEPC-treated water. Denature the RNA at 65 °C for 2 min to disrupt secondary structures, and then immediately place on ice.
2. Prepare magnetic beads/binding buffer suspension:
  - Transfer 270 μl of well resuspended Dynabeads™ magnetic beads to a 1.5 ml eppendorf tube. Place the tube on a DynaMag™-2 magnet stand for 30 s, or till all beads adhere to the tube wall, and then discard the supernatant.
  - Takeout the tube from the magnetic stand, and add 135 μl binding buffer to equilibrate the beads.
  - Put the tube back on the magnetic stand and discard the supernatant.
  - Takeout the tube from the stand, and add 135 μl binding buffer to the beads.
3. Poly(A) RNA isolation:
  - Mix the total RNA from step 1 and the beads/binding buffer suspension from step 2d.
  - Gently rotate the mixture using a roller for 5 min at room temperature to allow the poly(A) RNA annealing to the oligo (dT)_25_ on the beads.
  - Place the tube on the magnetic stand till the solution become clear, discard the supernatant.
  - Remove the tube from the stand and wash the poly(A) RNA-bead complex twice with 270 μl washing buffer B (carefully discard all traces of supernatant between each washing step).
  - Elute the poly(A) RNA from the beads by adding 13 μl of 10 mM Tris–HCl (pH 7.5). Keep the tube at 65 °C for 2 min and then place immediately on the magnetic stand.
  - Transfer 12 μl eluted mRNA to a new RNase-free 1.5 ml Eppendorf tube.

#### Ligation of 5′ RNA adapter

1. Add 1 μl 5′ RNA adaptor (100 μM) to 12 μl mRNA, mix and incubate the tube at 65 °C for 5 min to disrupt the secondary structure. Then place the tube on ice to chill (~ 2 min) and centrifuge briefly.
2. Add the following reagents to the poly(A) RNA/adaptor mixture, mix thoroughly by pipetting up and down and centrifuge briefly. When multiple samples are handled, prepare master mix by multiplying the number of samples and plus 10% extra, add 7 μl of the master mix to each poly(A) RNA/adaptor mixture.

**Table:** 

Reagents	Volume (μl)
T4 RNA ligase buffer	2
10 mM ATP	2
RNaseOut™ (40 U/μl)	1
T4 RNA ligase (5 U/μl)	2
Total	7

3. Incubate the ligation reaction at 37 °C for 1 h, and add 115 μl of DEPC-treated water to the ligation reaction and proceed immediately to the next step.

#### Purification of 5′RNA adapter ligated poly(A) RNA

Perform a 2nd round poly(A) RNA purification to remove the unincorporated 5′RNA adapter and purify 5′RNA adaptor ligated poly(A) RNA. To do this, repeat the steps described in section “Poly(A) RNA purification” with the exception that final mRNA is eluted in 26 μl of 10 mM Tris–HCl (pH 7.5). Transfer 25 μl RNA adaptor ligated poly(A) RNA to a thin-walled PCR tube.

#### First-stranded cDNA synthesis

SuperScript™ II reverse transcriptase is used to synthesize the 1st strand cDNA. SuperScript™ III and other reverse transcriptase can be used, and the components for the reverse transcription reaction should be adjusted accordingly.

1. Add dNTPs and RT primer to the adaptor ligated poly(A) RNA and mix well.

**Table:** 

Reagents	Volume (μl)
Adapter ligated mRNA	25
RT primer (100 μM)	2
dNTP mix (10 μM of each)	2
Total	29

2. Denature the mixture at 65 °C for 5 min to remove any RNA secondary structure, and then cool on ice.
3. Add 1st strand buffer, DTT and RNaseOUT, mix well and centrifuge briefly. Leave the tube at 42 °C for 2 min

**Table:** 

Reagents	Volume (μl)
5× 1st strand Buffer	10
0.1 M DTT	5
RNase OUT	2
Total	17

4. Add 4 μl SuperScript™ II reverse transcriptase, mix well and keep the tube at 42 °C for 1 h.
5. Incubate the reaction at 72 °C for 15 min.

#### First PCR amplification

Use Platinum^®^ Taq DNA Polymerase High Fidelity to prepare the 2nd strand cDNA.

1. Assemble the reaction in the following order.

**Table:** 

Reagents	Volume (μl)
cDNA	50
10× high fidelity PCR buffer	10
50 mM MgSO_4_	4
dNTPs (10 mM of each)	2
10 μM 5′ adaptor primer	2
10 μM 3′ adaptor primer	2
Platinum^®^ Taq DNA polymerase high fidelity	0.4
H_2_O	29.6
Total	100

2. Divide the PCR reaction into 3 thin-walled PCR tubes, and add 45 μl each into two tubes, and the remaining 10 μl into another tube (for experienced technicians, divide the PCR reaction into 2 tubes, 50 μl each, and omit the following step 4).
3. PCR amplification. PCR reaction conditions: 94 °C for 2 min, 94 °C for 30 s, 58 °C for 30 s, 72 °C for 5 min, 7 cycles total, 72 °C for 5 min, then hold at 4 °C. For 10 μl reaction, keep 15 PCR cycles.
4. Separate the 10 μl PCR reaction on a 1% agarose gel. If smear ranging from 500 to 2500 bp is visible (Additional file 1: Figure S1) which can indicate that the 5′RNA adaptor ligation and 1st strand cDNA synthesis worked well. Then proceed to the next step.

#### PCR product purification using MinElute PCR purification kit

PCR product is purified according to the procedure of MinElute PCR Purification using a microcentrifuge (QIAGEN).

1. Combine the PCR reaction (90 μl) with 5 times volume of Buffer PB (450 μl) containing pH indicator. If the color of the mixture is orange or violet, add 10 μl of 3 M sodium acetate, and mix well.
2. Place a MinElute column in a 2 ml collection tube, and transfer the PCR/PB buffer mixture to the MinElute column and centrifuge at maximum speed for 1 min at room temperature.
3. Discard flow-through, and wash column by adding 750 μl buffer PE and centrifuge at maximum speed for 1 min.
4. Discard flow-through and centrifuge the column for an additional 1 min at maximum speed.
5. Put the column into a new 1.5 ml Eppendorf tube, add 12 μl water at the center of the membrane, let the column stand for 1 min, and then centrifuge for 1 min, repeat this step again. Transfer 22 μl elution to a new tube.

#### Digestion with EcoP15I

Set up the digestion reaction in the following sequence:

**Table:** 

Reagents	Volume (μl)
PCR product	22
10× NEB buffer 3.1	3
10 mM ATP	3
10 U/μl EcoP15I	2
Total	30

1. Incubate the digestion at 37 °C for 1–2 h.
2. After digestion, inactivate EcoP15I at 65 °C for 20 min, and then cool the digested mixture at room temperature (do not place the tube on ice). Proceed immediately to the next step.

#### 3′double-strand DNA (dsDNA) adapter ligation

1. Prepare dsDNA adaptor. Mix equal amount of dsDNA_top and dsDNA_bottom oligos as shown in the table below. Mix well and centrifuge briefly and heat the mixture for 5 min at 100 °C and leave the tube at room temperature until it cools down. The dsDNA adapter should be prepared freshly each time, and this can be done during EcoP15I digestion.

**Table:** 

Reagents	Volume (μl)
dsDNA_top (100 μM)	10
dsDNA_bottom (100 μM)	10
Total	20

2. Double strand DNA adaptor ligation. Set up the ligation mixture in the following order, mix well, centrifuge briefly and leave the ligation reaction at room temperature for 1 h.

**Table:** 

Reagents	Volume (μl)
EcoP15I digestion	30
5× ligase buffer	12
dsDNA adaptor	3
T4 DNA ligase (1 U/μl)	2
H_2_O	13
Total	60

#### PAGE purification of ligated dsDNA products (79 bp)

1. Prepare 12% non-denaturing PAGE-TBE gel mixture in a 50 ml conical vial in the following order. Then add 75 μl of freshly prepared 10% ammonium persulfate (APS) and 15 μl tetramethyl ethylene diamine (TEMED), mix well and cast a PAGE gel with 1.5 mm spacer. Prepare the PAGE gel during dsDNA adaptor ligation.

**Table:** 

Reagents	Volume (for 1 gel) (ml)
40% acrylamide stock (ml)	4.5
5× TBE	1.5
H_2_O	8.91
Total	15

2. Prepare 0.5× TBE buffer.
3. Add 12 μl 6× gel loading buffer to the ligation reaction, mix well and load samples in two wells. Leave 1 empty well between different samples if multiple samples are handled.
4. Load 20 bp, 50 bp DNA ladder at both sides of the samples.
5. Run gel in 0.5× TBE buffer till good separation (160 V, 1 h).
6. While running the gel, prepare 0.5 ml tubes by puncturing one hole with a 21-gauge (21 G) needle at the bottom, and place the tubes inside 2 ml tubes.
7. Remove gel carefully and stain with 50 ml of 1× SYBR gold in 0.5× TBE for 5 min by slowly shaking.
8. Visualize gel on a UV transilluminator. The ligated products should have a size of 79 bp (79 bp = 5′RNA adapter (31 bp) + EcoP15I-digested tag (27 bp) + 3′ dsDNA adapter (21 bp), but, the ligation band is not visible at this step, therefore, cut gel area corresponding to DNA ladder size between 70 and 90 bp and put it into a 0.5 ml tube with a hole (Fig. 2).

**Fig. 2:**
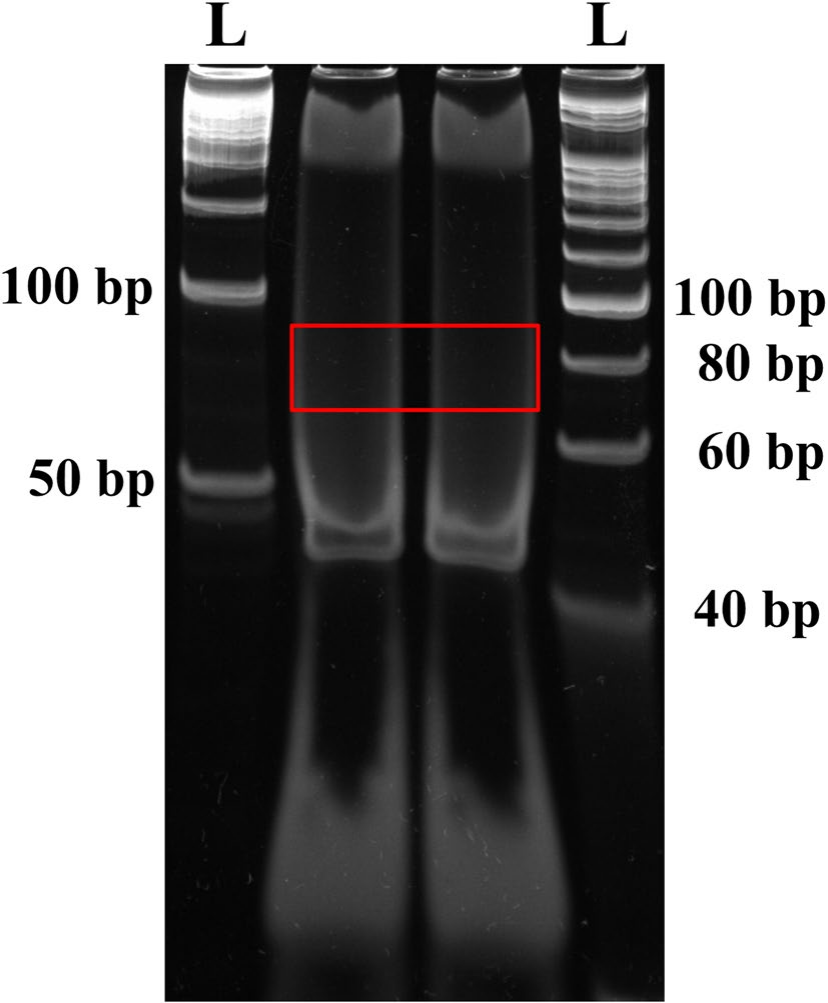
Purification of the 3′dsDNA adaptor ligated products (79 bp) on a PAGE gel. The gel pieces corresponding to 70–90 bp DNA fragment was isolated and eluted. The letter ‘L’ denotes the DNA ladder
9. Centrifuge the gel pieces for 2 min at maximum speed; make sure all of gel pieces are in 2 ml tube. Otherwise, puncture more holes in the 0.5 ml tube and spin 1 min again.
10. Remove the 0.5 ml tube and add 400 μl H_2_O to the 2 ml tube.
11. Elute the ligation fragments overnight at 4 °C with gentle agitation.

### Days 2 and 3

#### Concentrate the dsDNA adaptor ligated products by ethanol precipitation

1. Transfer the entire elution sample (suspension with gel) into a COSTAR Spin-X column centrifuge filter, spin at 12,000×*g* for 2 min. Make sure all of the liquid spins out, and spin longer if necessary.
2. Measure the liquid volume, add 10% volume of 3 M sodium acetate (NaOAc, pH 5.5), 2 volumes of 100% ethanol and 1 μl glycogen. Mix well and leave the tube at − 80 °C for 2–3 h.
3. Centrifuge at 12,000×*g* for 30 min at 4 °C.
4. Discard supernatant, and wash the pellet with 70% ethanol, and centrifuge at 12,000×*g* for 5 min at 4 °C.
5. Discard supernatant carefully and dry the pellet for 5 min at room temperature.
6. Dissolve the pellet in 40 μl H_2_O.
7. Transfer 39.8 μl ligation product to a new thin wall PCR tube.

#### PCR enrichment of degradome library

1. Prepare PCR reaction in the following order.

**Table:** 

Reagents	Volume (μl)
Ligation product	39.8
10× high fidelity PCR buffer	5
50 mM MgSO_4_	2
dNTPs (10 mM each)	1
10 μM RP1	1
10 μM 3′ index primer	1
Platinum^®^ Taq DNA polymerase high fidelity	0.2
Total	50

2. Run PCR cycle: 94 °C for 2 min, 94 °C for 30 s, 60 °C for 30 s, 72 °C for 30 s, 11–15 cycles, 72 °C for 5 min, then hold at 4 °C.

#### PAGE purification of the final PCR products

1. Prepare 8% non-denaturing PAGE gel (this can be done during the PCR amplification step). Prepare the gel mix in a 50 ml conical vial in the following order. Then add 75 μl freshly prepared 10% APS and 15 μl of TEMED. Mix well and cast a PAGE gel with 1.5 mm spacer.

**Table:** 

Reagents	Volume (for 1 gel) (ml)
40% acrylamide stock	3
5× TBE	1.5
H_2_O	10.41
Total	14.91

2. Add 10 μl of 6× gel loading buffer to the final PCR reaction and load the PCR reaction into two wells. Meanwhile, load 50 bp, 1 kb DNA ladder at left and right side of the samples.
3. Run gel in 0.5× TBE buffer till good separation (120 V, 1 h).
4. While running the gel, prepare 0.5 ml tubes by puncturing one hole with a 21-gauge (21 G) needle at the bottom, and place it inside the 2 ml tubes.
5. Remove gel carefully and stain the gel using 50 ml of 0.5× TBE containing ethidium bromide for 5–10 min.
6. Visualize gel on transilluminator. The final PCR product should have a clear band near 150 bp DNA marker (Fig. 3a). Excise the PCR product band and put the gel pieces into the punctured 0.5 ml tube.

**Fig. 3:**
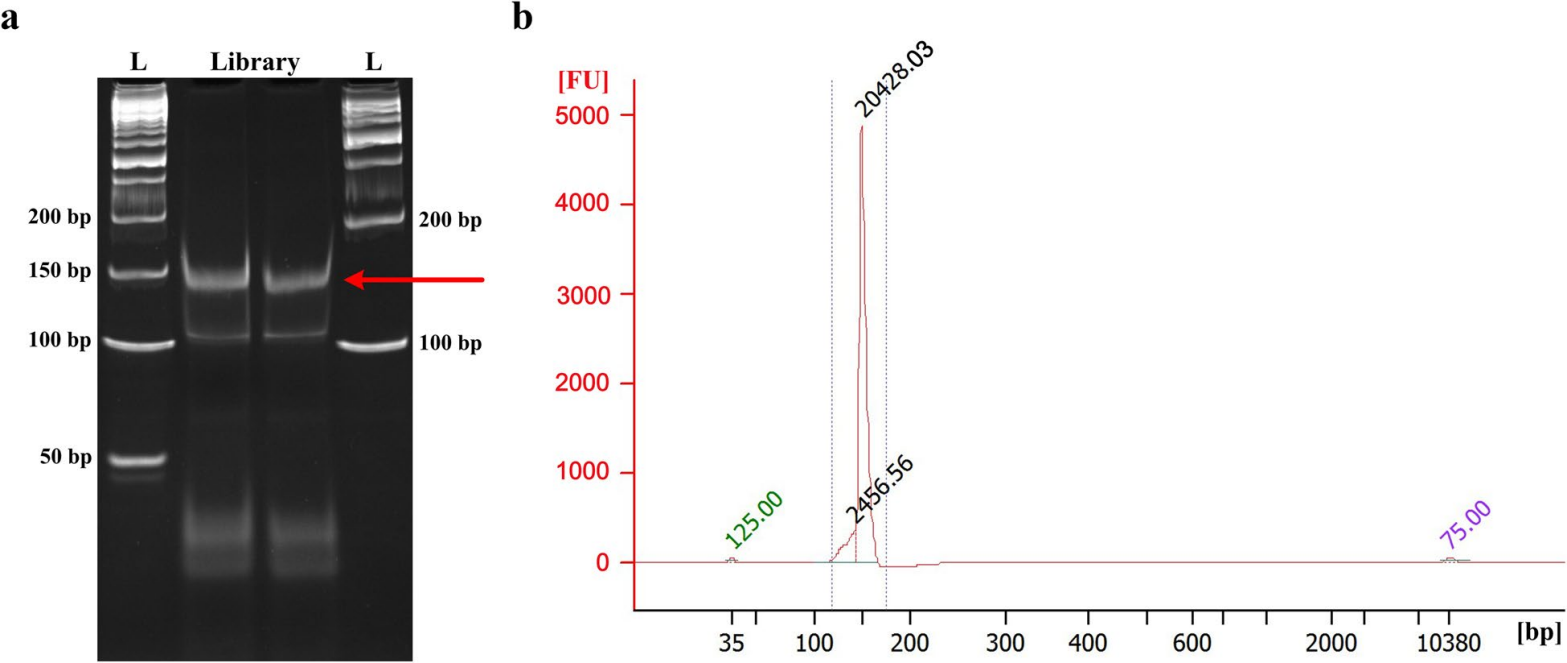
Degradome library purification and quality assessment. **a** PAGE purification of the final PCR products (~ 150 bp). The letter ‘L’ denotes the DNA ladder. **b** Determination of fragment size profile of the degradome library using Agilent Bioanalyzer high sensitivity DNA chip. A clear peak at ~ 150 bp but no other peaks should be visible
7. Centrifuge the gel pieces for 2 min at maximum speed; make sure all of the gel pieces are in 2 ml tube.
8. Discard the 0.5 ml tube and add 400 μl H_2_O to the 2 ml tube.
9. Elute the degradome library overnight at 4 °C with gentle agitation.
10. Repeat the same precipitation procedure as step “Concentrate the dsDNA adaptor ligated products by ethanol precipitation” with the exception that the final pellet is dissolved in 15 μl nuclease-free water.

#### Quality assessment of degradome library and Illumina sequencing

1. Determine the fragment size and purity of the degradome library using an Agilent Bioanalyzer High Sensitivity DNA chip. Optimal degradome library should have a tight fragment around 150 bp (Fig. 3b).
2. Determine degradome library concentration by fluorometry (Qubit High Sensitivity Kit or Picogreen).
3. High throughput sequencing of degradome library. The degradome library prepared using this method can be treated as small RNA library for sequencing with single-end 50 nt reads. Several degradome libraries can be pooled and multiplexed, like small RNA libraries.

## Results and discussion

**Fig. 4:**
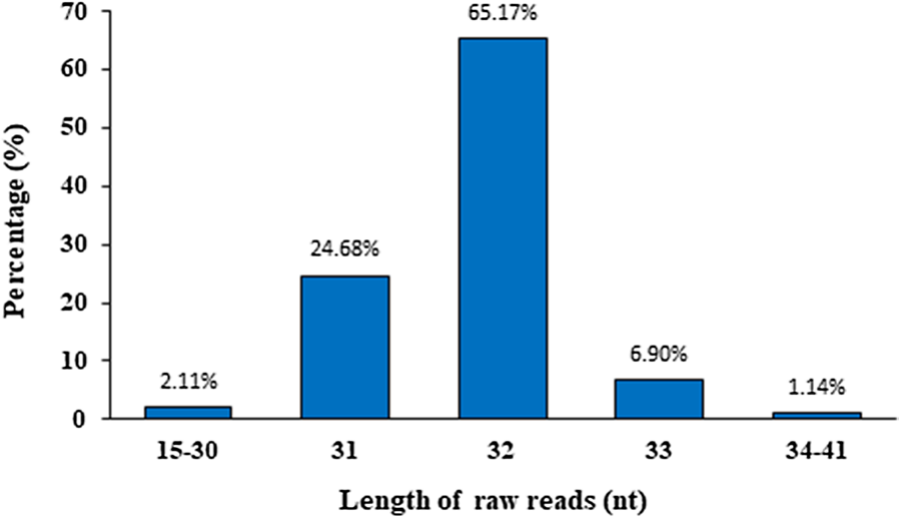
Size distribution of the raw data generated from a rice degradome library

**Fig. 5:**
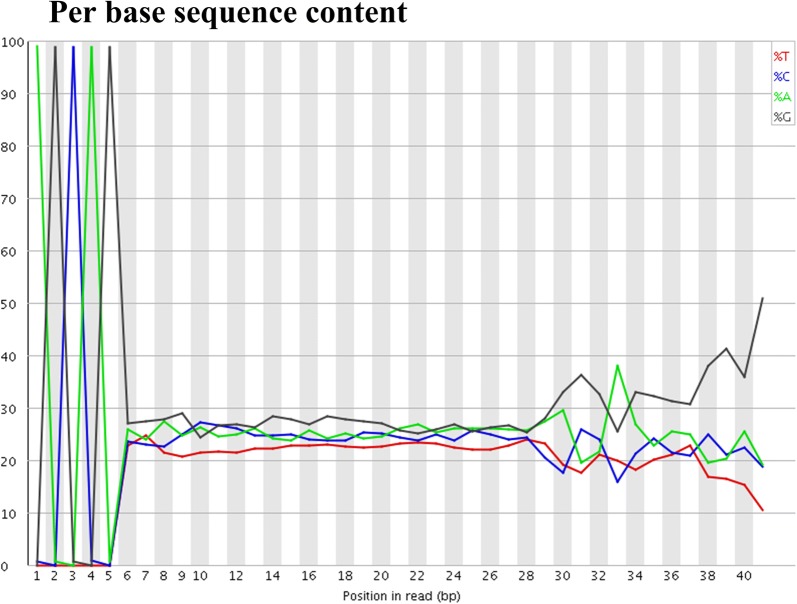
Per base sequence content of the raw reads from a rice degradome library. “AGCAG” is the signature sequence derived from 5′RNA adaptor and should be trimmed prior to the bioinformatics analysis

We aimed to improve the method for generating degradome libraries that can be easily sequenced using Illumina sequencer and can also yield longer read lengths. We generated degradome libraries of expected size of 150 bp (Fig. 3). Using the small RNA library sequencing approach, we sequenced our degradome libraries that were of high quality (Additional file 2: Figure S2). The majority raw reads were 32-nt long, consisting of tag size of 27-nt, followed by 31- and 33-nt long raw reads, containing tags of 26-nt and 28-nt, respectively (Fig. 4). We further examined quality of raw reads, and 99% raw reads began with “AGCAG” (Fig. 5), which is derived from the nucleotides added to the 3′end of 5′RNA adaptor for generation of Ecop15I recognition site. The signature of “AGCAG” in raw reads, together with 95.75% raw reads of 31–33 nt long (Fig. 4), indicate the feasibility of usage of EcoP15I in degradome library generation. To identify plant miRNA targets, degradome data generated using this method can be analyzed using CleaveLand [22] or SeqTar [23] programs. The “AGCAG” signature need be trimmed from the raw reads prior to analyzing of the degradome reads.

**Fig. 6:**
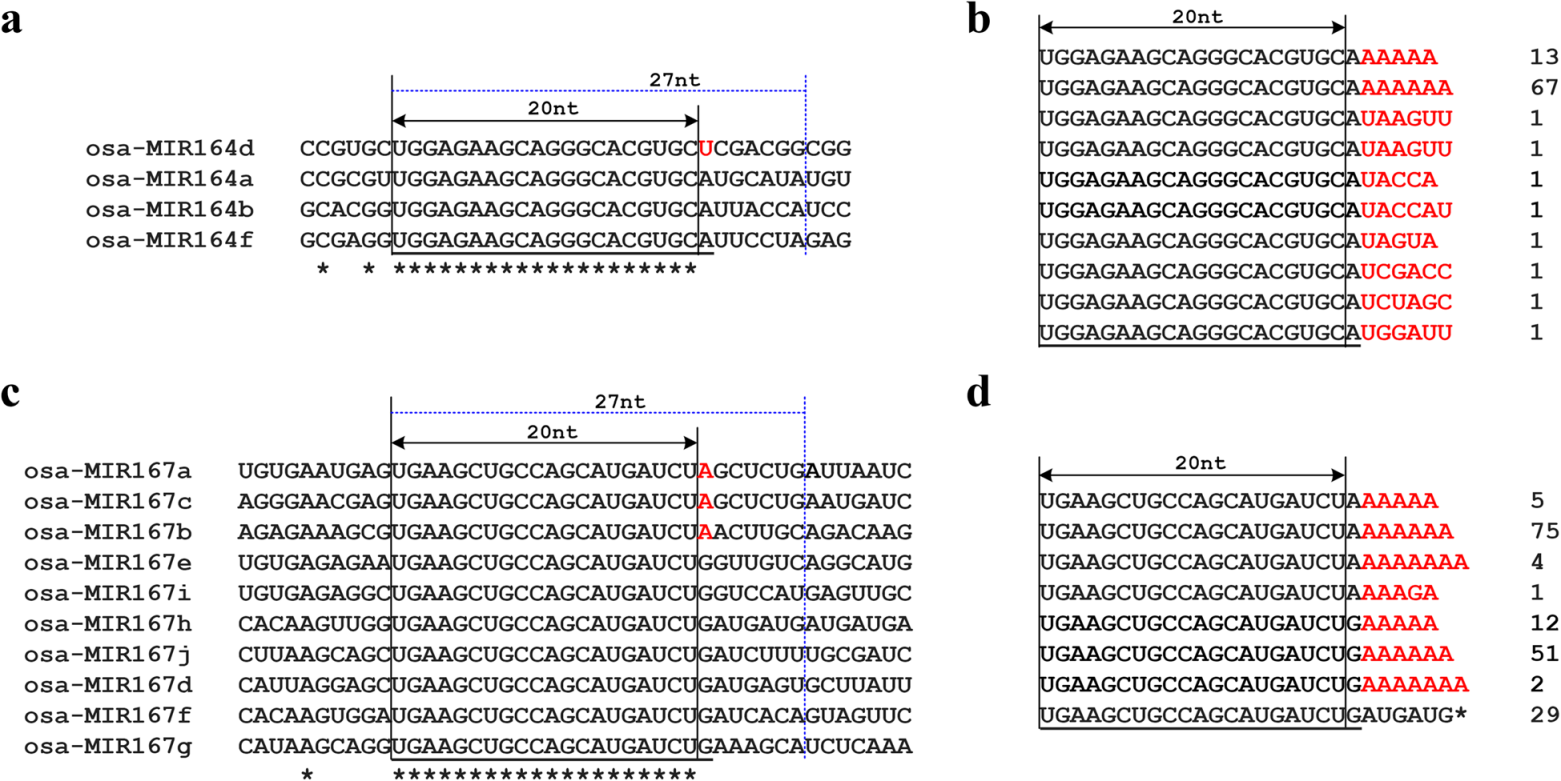
The modified method can improve the mapping accuracy of the sequencing reads. **a**, **c** Alignment of partial rice miR164 and miR167 family precursors (Red letters denote different nucleotides among these miRNA members). The 20-nt tags generated using previous method can be mapped to multiple genes, while the 27-nt tags generated from these genes using the present method can distinguish those differences easily. **b**, **d** Tag sequences and frequency obtained from the modified method which contain mature miR164 and miR167 sequence (red letters denote detected nucleotides at mature miRNA end, and the sequence with * indicates this tag derived from miR167 h precursor. Mature miRNA sequences are underlined)

Tags corresponding to mature miRNAs have been reported in Arabidopsis, Rice, moss and mouse [8, 13, 19, 20]. Using SeqTar pipeline [23], the degradome data from our previous study [8] and the present study was aligned to the precursors of the 22 evolutionary conserved miRNA families (miR156, miR159, miR160, miR162, miR164, miR166, miR167, miR168, miR169, miR171, miR172, miR319, miR390, miR393, miR394, miR395, miR396, miR397, miR398, miR399, miR408, and miR444). Sequence alignment of the 20-nt tags revealed that 48 precursors (32%) had more than 5 reads exactly mapped to the beginning sites of miRNA-5p, and many tags could be mapped to multiple mature miRNAs belonging to the same miRNA family, although it is unknown whether these tags were derived from the adenylated miRNAs or incomplete DCL1 cleavage during miRNA biogenesis. Similar mapping of the rice degradome data generated in this study showed that only precursors of miR167h, miR168a and miR169i have tags more than 5 reads (30, 38 and 22 reads, respectively) mapped to the beginning sites of miRNA-5p. We further analyzed the origin of the 20-nt tags mapped to mature miRNAs using the degradome data generated in this study, the outcome showed that the incomplete DCL1 cleavage on miRNA precursors is not common in rice. A 20-nt tag of TGCCTGGCTCCCTGTATGCC with 52 reads could be simultaneously mapped to the beginning site of miR164a, b, d and f (Fig. 6a, Additional file 3: Figure S3). If this tag was generated from DCL1 incomplete cleavage during miRNA biogenesis, the corresponding 27-nt tags from precursors of miR164a, b, d and f will be different from each other (Fig. 6a) and no such mapped tags were found in the 27-nt degradome data; if this tag was derived from miRNA164 adenylation, the corresponding 27-nt tags generated using this modified method cannot be mapped to the miR164 precursors. Indeed, we found 27-nt tags containing the 20-nt tag TGCCTGGCTCCCTGTATGCC which were largely derived from miR164 adenylation (Fig. 6b). Similarly, a 20-nt tag of TGAAGCTGCCAGCATGATCT with a frequency of 25 reads could be mapped to the beginning sites of miR167a, b, c, d, e, f, g, h, i and j (Fig. 6c, Additional file 4: Figure S4). Using the present method, we found that this tag can be generated from not only miRNA167 adenylation, but also from the incomplete cleavage of rice miR167h precursor (Fig. 6d). These results clearly demonstrate that the 27-nt tags generated by the modified method can enhance the mapping accuracy of the reads.

Compared with the previous PARE protocol [18], the modifications included in this protocol are as follows: (1) altered 5′RNA adaptor: 5′RNA adaptor sequence in the previous protocol is 5′GUUCAGAGUUCUACAGUCCGAC-3′, which contains MmeI recognition site (underlined), and our modified 5′RNA adaptor sequence is 5′GUUCAGAGUUCUACAGUCCGAC*GAU*C*AGCAG* 3′, which is longer (italics) than previous adaptor and contains the additional recognition site of EcoP15I (italics and underlined). (2) Agencourt^®^ AMPure^®^ XP (Beckman-Coulter) is convenient to purify 1st round PCR product when multiple PARE libraries are constructed, but we used MinElute^®^ PCR purification kit (QIAGEN) to purify, which is quick and convenient for purifying PCR products when only a few samples are handled. Other brand PCR purification kits should work well too. (3) altered 3′dsDNA adapter: previously used top sequence: 5′ TGGAATTCTCGGGTGCCAAGG 3′, and bottom: 5′ CCTTGGCACCCGAGAATTCCANN 3′; while the altered 3′ dsDNA adapter sequences are as follows (top) 5′ NNTGGAATTCTCGGGTGCCAAGG 3′, and (bottom) 5′ CCTTGGCACCCGAGAATTCCA 3′. (4) altered final 5′ PCR primer: previously used primer sequence is 5′ AATGATACGGCGACCACCGACAGGTTCAGAGTTCTACAGTCCGA 3′, however, RP1 from TruSeq^®^ Small RNA Sample Prep Kit is used as final 5′ primer in this protocol. (5) previous PARE method generates degradome libraries of 128 bp with tags of 20-nt, whereas this method generates the final libraries of 150 bp with tags of 26- to 28-nt, mainly 27-nt. (6) Illumina HiSeq sequencing of PARE library prepared by previous method must use PARE specific sequencing primer: 5′ CCACCGACAGGTTCAGAGTTCTACAGTCCGAC 3′; The degradome library generated using this modified method can be sequenced in the same way as small RNA library, which is easier and more convenient. Therefore, degradome libraries generated using present method can even be pooled with small RNA libraries for sequencing. Even if the same index is used in both the libraries, i.e., degradome library and small RNA library, these libraries can still be pooled for sequencing, because degradome reads contain the “AGCAG” sequence signature that can be used to distinguish reads derived from degradome library rather than from small RNA library.

## Conclusions

Here, we present a modified protocol for construction of degradome libraries, which can be used for studying degraded mRNAs with free 5′ monophosphates and poly(A) tail. Like previous methods [18], the entire protocol can be completed within 3 days. However, due to the introduction of EcoP15I recognition site at the 3′end of 5′RNA adaptor of TruSeq small RNA library (RA5), the generated tag is ~ 27-nt long. This facilitates a better accuracy in mapping the reads. The introduced modifications allow the libraries to be sequenced as Illumina TruSeq library. The degradome libraries can even be pooled with small RNA libraries for sequencing, which is convenient for analyzing both small RNAs and their targets simultaneously. The tags derived from miRNA precursor processing intermediate differ from tags of miRNA/miRNA* adenylation, therefore, this method can also be used for gaining insights into miRNA biogenesis.

## Supplementary information

**Additional file 1: Figure S1.** Visualization of the 1st PCR product. PCR product was separated using 1% agarose gel. The letter ‘L’ denotes the DNA ladder.

**Additional file 2: Figure S2.** Quality scores across all bases of a rice degradome library raw data.

**Additional file 3: Figure S3.** Signature abundance throughout the length of rice miR164 precursors. The 20-nt tags generated by previous method [8] were plotted on to rice miR164 precursors; miRNA precursor sequences were extended 50-nt at 5′ and 3′ end, respectively. Arrows indicate the beginning site of miR164.

**Additional file 4: Figure S4.** Signature abundance throughout the length of rice miR167 precursors. The 20-nt tags generated by previous method [8] were plotted on to the rice miR167 precursors; miRNA precursor sequences were extended 50-nt at 5′ and 3′ end, respectively. Arrows indicate the beginning site of miR167.

## Data Availability

The degradome data obtained in this study was deposited at the National Center for Biotechnology Information Gene Expression Omnibus (NCBI, GEO, http://www.ncbi.nlm.gov.geo/) under accession number GSE138545.
