# Supplementary figures and images for: An improved method of constructing degradome library suitable for sequencing using Illumina platform
Source: Plant Methods. 2019 Nov 18;15:134. doi: 10.1186/s13007-019-0524-7 (PMC6859640; doi:10.1186/s13007-019-0524-7)

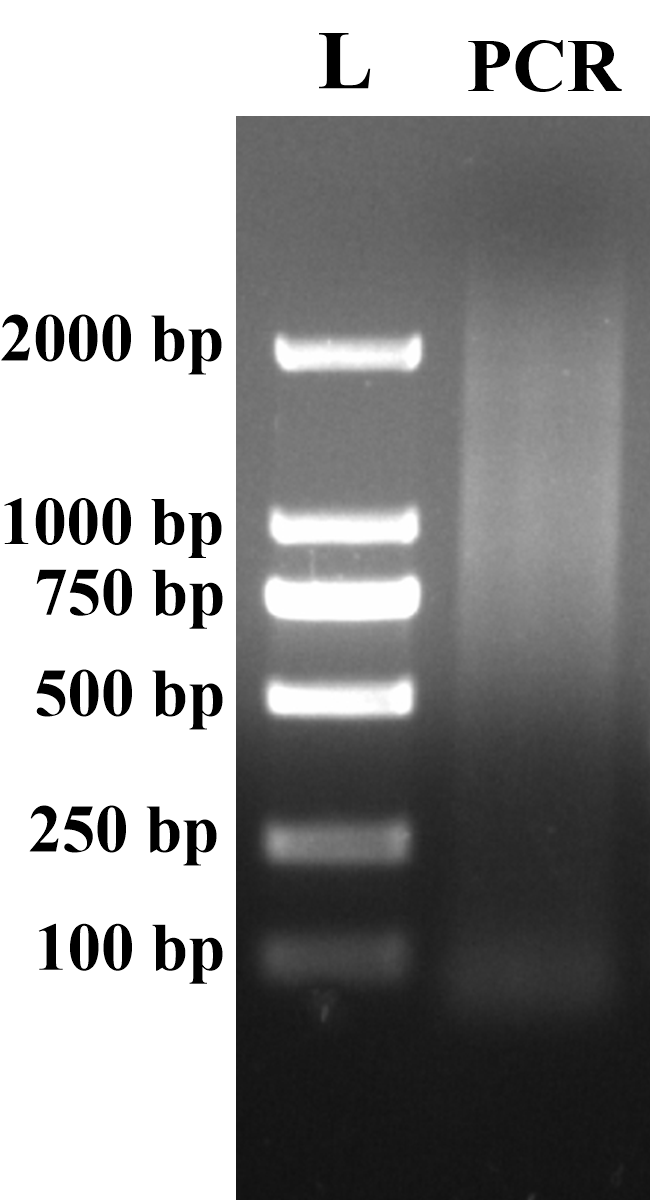

Supplement: Supplementary file 1 — Additional file 1: Figure S1. Visualization of the 1st PCR product. PCR product was separated using 1% agarose gel. The letter ‘L’ denotes the DNA ladder. [file 13007_2019_524_MOESM1_ESM.tif]

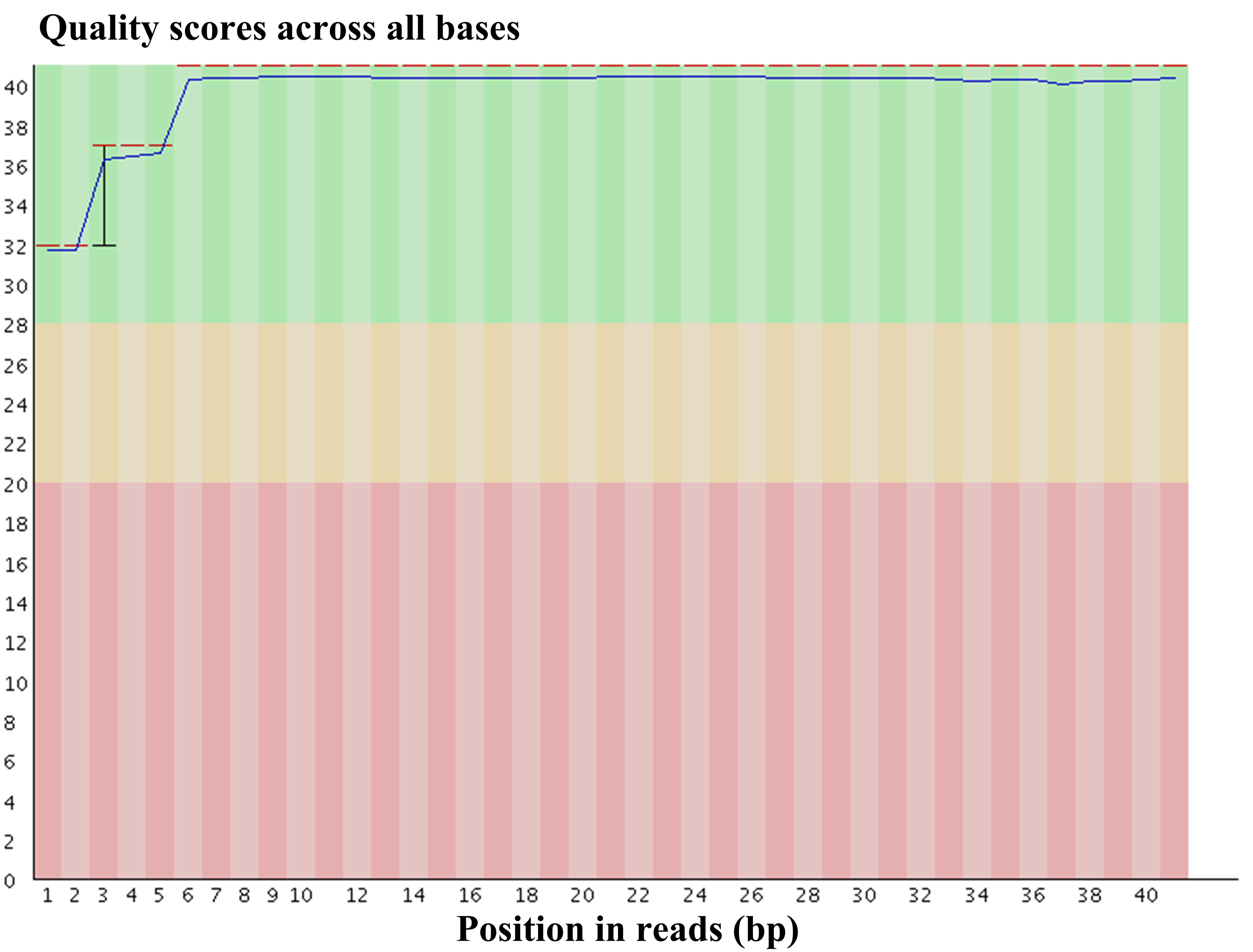

Supplement: Supplementary file 2 — Additional file 2: Figure S2. Quality scores across all bases of a rice degradome library raw data. [file 13007_2019_524_MOESM2_ESM.tif]

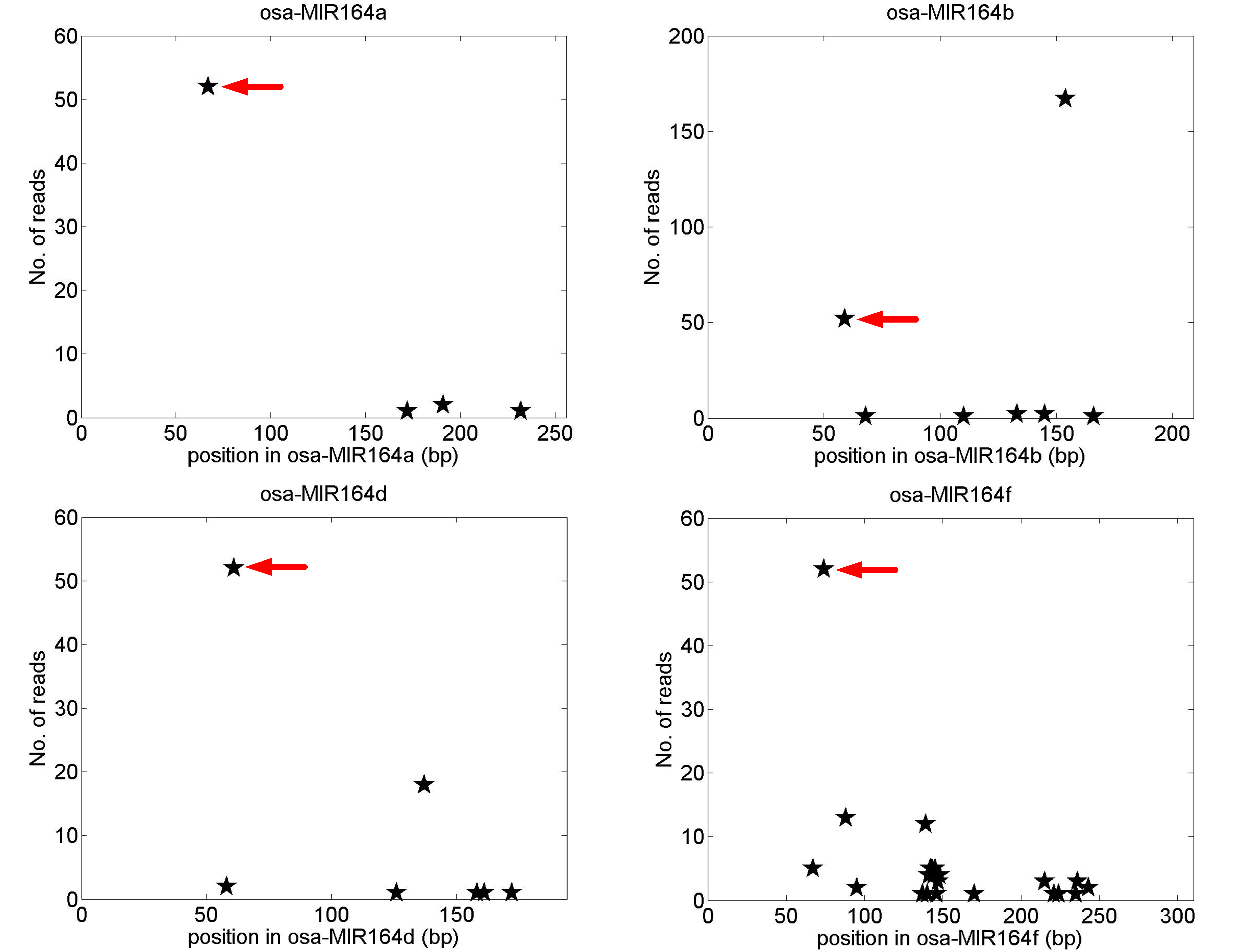

Supplement: Supplementary file 3 — Additional file 3: Figure S3. Signature abundance throughout the length of rice miR164 precursors. The 20-nt tags generated by previous method [8] were plotted on to rice miR164 precursors; miRNA precursor sequences were extended 50-nt at 5′ and 3′ end, respectively. Arrows indicate the beginning site of miR164. [file 13007_2019_524_MOESM3_ESM.tif]
